# Inverse correlation between acid reflux and dwell time of voice protheses (Provox^®^) in laryngectomy/laryngopharyngectomy patients: a retrospective analysis of a prospective cohort

**DOI:** 10.1007/s00405-026-10370-1

**Published:** 2026-06-10

**Authors:** Daniel Runggaldier, Baselt Bastian, Naomi Iob, Cosima Riemenschnitter, Christian Thüring, Jérôme R. Lechien, Jérôme Bonzon, Jörg E. Bohlender, Grégoire B. Morand

**Affiliations:** 1 Department of Otorhinolaryngology, Head & Neck Surgery, University Hospital Zurich, University of Zurich, Zürich, Switzerland; 2 Department of Otorhinolaryngology, Division of Phoniatrics and Speech Pathology, University Hospital Zurich, University of Zurich, Zurich, Switzerland; 3 University of Zurich, Zurich, Switzerland; 4 Department of Surgery, Research Institute for Language Science and Technology, University of Mons, Mons, Belgium; 5 Department of Clinical Pharmacology and Toxicology, University Hospital Zurich, University of Zurich, Zürich, Switzerland

**Keywords:** Provox voice prosthesis, Laryngectomy, Extraesophageal reflux, Proton pump inhibitors, PPI

## Abstract

**Objectives:**

To evaluate the impact of extraesophageal acidic reflux and proton pump inhibitor (PPI) therapy on the dwell time of Provox^®^ voice prostheses.

**Methods:**

This is a retrospective review of prospectively recruited patients with total laryngectomy/ laryngopharyngectomy and rehabilitation with Provox^®^ voice prosthesis who underwent 24 h pH monitoring study. Associations between multiple acid exposure metrics (percentage of time at pH < 6.5, < 6.0, < 5.5, < 5.0, and Ryan score), clincal history and mean dwell time of the last three voice prostheses were assessed using linear regression analysis.

**Results:**

Twenty-two patients were included. Regression analysis showed an inverse relationship between acid exposure and the average dwell time in days of the voice prostheses (β = −1.85, 95% CI: −2.90 to −0.80, *p* = 0.0014, R2 = 0.40 for pH < 6.5 resp. β = −1.74, 95% CI: −2.95 to −0.52, *p* = 0.0071, R2 = 0.30 for pH < 6.0). Twelve patients were on regular PPI treatment, 10 patients were off PPI. Patients with PPI usage did not show a significantly different acid exposure in the neopharynx. No difference in the dwell time between the patients with and without regular PPI therapy was observed.

**Conclusion:**

In our cohort, acid reflux exposure is inversely correlated with the dwell time of Provox^®^ voice prostheses. PPI treatment was not associated with a reduced acid exposure and prolonged dwell time of the voice prostheses, indicating insufficient effect or other mechanisms involved. Further studies are needed to identify effective therapeutic strategies in prolonging voice prostheses dwell times.

**Supplementary Information:**

The online version contains supplementary material available at https://doi.org/10.1007/s00405-026-10370-1.

## Introduction

Voice rehabilitation after laryngectomy or laryngopharyngectomy is traditionally done in three ways: esophageal speech, electrolarynx and nowadays most commonly tracheooesophageal fistula with a voice prosthesis [2, 15, 16, 22]. There are two main manufacturers of voice prostheses on the Western market: Atos for Provox^®^ and the Blom Singer^®^ prostheses. At the university hospital of Zurich, the vast majority of patients are rehabilitated with Provox Vega^®^ prostheses.

In daily clinical practice, there is a great discrepancy in the dwell time of each Provox^®^ prosthesis, with a range of patients requiring a change every few weeks to not requiring a change for over years [2]. Frequent changes of defect voice prostheses, that are often leaking and hence result in aspirations, are not only burdensome for the patient, but also increase health care costs. It can also lead to dreadful complications such as enlargement of the tracheooesopahgeal fistula, which is difficult to manage with or without surgery [2, 20].

Factors leading to this discrepancy are not fully elucidated. Some evidence exists that fungal colonization and radio(chemo)therapy could be detrimental to the dwell time of a voice prosthesis [4, 12]. Due to the resection of the upper esophageal sphincter (cricopharyngeal muscle) in total laryngectomy, patients might be exposed to a higher extend of gastric reflux. There are already scarce data on the prevalence and impact of reflux disease on the dwell time of a voice prosthesis, but studies being inconsistent and conflicting [4, 6, 7, 14, 18].

Hence, the aim of our study was to retrospectively analyze data of a prospective cohort of total laryngectomy/laryngopharyngectomy patients rehabilitated with a Provox^®^ voice prosthesis, who underwent 24 h pH monitoring study of the neopharynx (Restech^®^). In order to investigate the effect of acid reflux on the lifetime of the voice prostheses, we correlated the dwell time with the acid exposure of the Provox^®^ voice prostheses.

## Materials and methods

### Study cohort

A retrospective, single institutional analysis of prospectively collected data was conducted. All patients presenting for routine follow-up after total laryngectomy or laryngopharyngectomy (generally with cricopharyngomyotomy) with tracheooesophageal fistula and Provox^®^ voice prosthesis were prospectively and consecutively recruited to undergo 24 h pH monitoring (Restech^®^) as part of their clinical workup between January 2023 and June 2025. pH monitoring was not prompted by reflux symptoms.Exclusion criteria were patients with Blom Singer^®^, patients with highly altered anatomical situations such as severe stenosis in the neopharynx that prevented proper placement of the pH monitoring probe, patients with pH monitoring study performed during hospitalization (due to higher proportion of supine position during hospitalization compared to normal everyday life), patients under 18 years of age at diagnosis.

Other data such as clinical history, medications (especially anti-reflux medication), and the mean dwell time of the last three voice prostheses prior to pH monitoring were collected from medical reports. The mean dwell time of the three most recent prostheses was used as a proxy for individual baseline prosthesis durability.Patients on regular proton pump inhibitor (PPI) treatment were not withdrawn from the PPI during pH monitoring study - as our aim was to capture the everyday acid exposure at the prosthesis level under long term PPI therapy (rather than the baseline acid exposure without PPI if the long term therapy is abruptly stopped). As a secondary outcome, differences in the dwell time of voice prostheses depending on PPI usage were assessed. Additionally, a comprehensive review of the medication as well as, if available, genotyping information for relevant pharmacogenes was performed by a board-certified specialist in clinical pharmacology and toxicology of the University Hospital of Zurich to rule out drug-drug interactions or other reasons for possible PPI ineffectiveness.

## Measurements

**Fig. 1:**
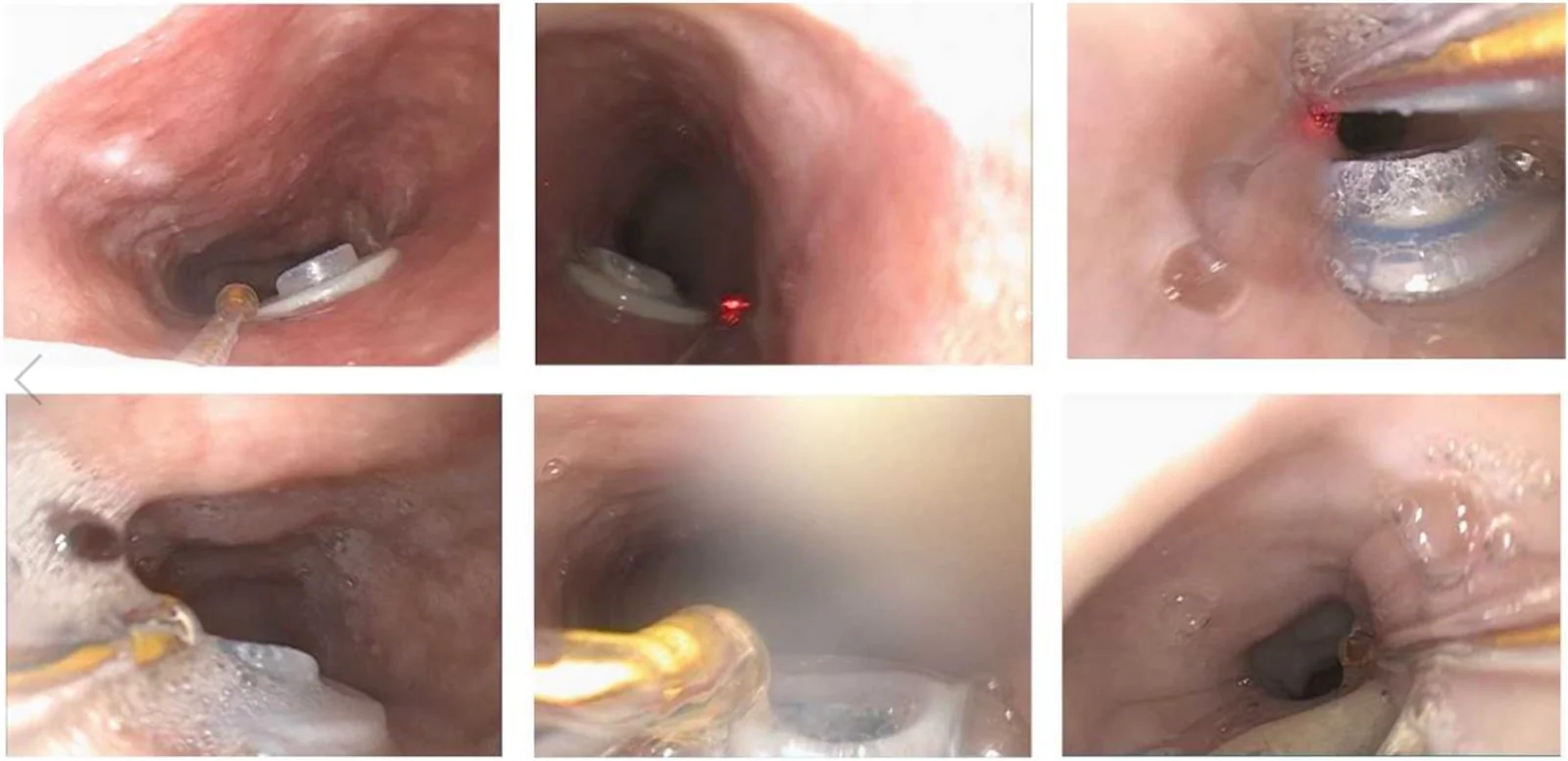
24h pH measurement (Restech) in the neopharynx at the level of the provox voice prosthesis. Placement of pH probe was controlled by transnasal fiberendoscopy and 6 examples of such probe placements are shown here

For pH monitoring, the pH probe was placed at the level of the voice prosthesis in the neopharynx/proximal esophagus as shown in Fig. 1 and the pH was measured for 24 h to assess acid exposure of the voice prosthesis. Percentage of monitoring time with pH dropping below 6.5, 6.0, 5.5, and 5.0, as well as the Ryan score in supine and upright position, were determined by Restech Data View^®^ software. The Ryan score is calculated based severe and very severe acid reflux events with pH dropping below 5.5 in upright and below 5 in supine position, duration of the longest reflux episode and reflux episodes in % of time [5, 17].

The primary outcome of the study was to assess for a correlation between acid exposure (as measured by 24 h pH monitoring) and dwell time of the voice prothesis. Secondary outcome was to assess for potential protective effect of PPI on the dwell time of voice prothesis, controlling for potential relevant pharmokinetics issues.

### Statistical analysis

Statistical analyses were conducted using R software (version 4.4.2). Patients’ characteristics and outcome variables are summarized as counts and percentages for categorical variables, and as medians with interquartile ranges (IQRs) for continuous variables. Linear regression analysis was used to assess associations between acid exposure metrics and average dwell time. To assess the robustness of the primary estimates, three complementary sensitivity analyses were performed: (1) robust regression using the Huber loss function to reduce the influence of outlying observations without excluding them, (2) Ordinary Least Squares regression (OLS) after exclusion of influential points identified by Cook’s distance > 1, and (3) OLS regression with log-transformation of the dependent variable (average dwell time) to address potential residual skewness. Influence diagnostics are reported in the Supplementary Appendix. Results are reported as regression coefficients (β), standard errors, 95% confidence intervals, p-values for the slope, and coefficients of determination (R²). The Wilcoxon rank-sum test was used to compare pH levels and average dwell time between patients with and without PPI use, as well as between patients with high and low Ryan scores.

Multivariable regression was not performed given the limited sample size (*n* = 22); the rationale and implications are addressed in the Discussion.

Approval from the corresponding ethics committee of the University of Zurich was obtained for our retrospective study (protocol number 2024–02159).

## Results

### Baseline characteristics

Twenty-two patients post laryngectomy or laryngopharyngectomy rehabilitated with a Provox^®^ voice prosthesis and who underwent pH monitoring study of the neopharynx, were included in the study. The median patient age was 73 years (IQR 63–76). The median time from laryngectomy to pH measurement was 3.5 years (IQR 1.5–8.1), and the median time from completion of radiotherapy to pH measurement was 6.8 years (IQR 3.6–13.2). The patient cohort contained 86% males and 14% females. Almost all patients undergone radio(chemo)therapy either before or after laryngectomy (Table 1).

**Table 1:** Demographics

Characteristics		*n* = 22
Age, years		73 (63, 76)
Sex Male Female		19 (86%) 3 (14%)
Radio(chemo)therapy No Yes		1 (4.5%) 21 (95%)
Time after Surgery Radiotherapy		3.5 (1.5, 8.1) 6.8 (3.6, 13.2)
Voice prosthesis Provox Vega Provox Vega XtraSeal Provox ActiValve		17 (77%) 3 (14.5%) 2 (9%)

Data are n (%) or median (IQR)

Most patients were rehabilitated with Provox Vega^®^, with a few cases with Provox Xtra Seal^®^ or Provox ActiValve^®^ prostheses (Table 1).

### Primary outcome

#### All prostheses types

**Fig. 2:**
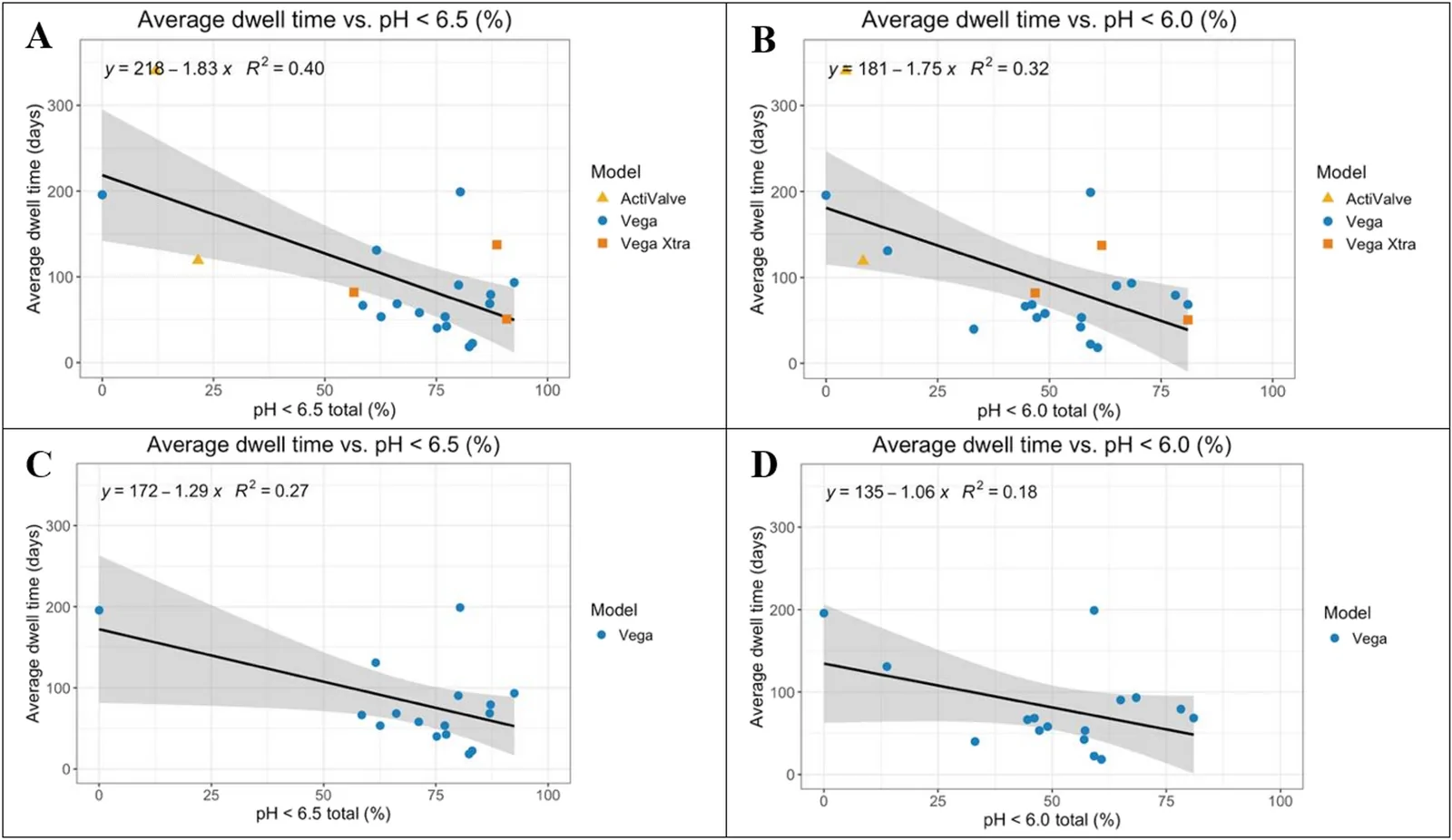
Regression analysis over all types of voice prostheses shows an inverse relationship between acid exposure (as assessed by the percentage of time in a 24-hour monitoring interval with pH dropping below 6.5 resp. 6) and the average dwell time in days of the voice prostheses. 2c and d: Regression analysis considering only classical provox vega voice prostheses also show an inverse relationship between acid exposure (as assessed by the percentage of time in a 24-hour monitoring interval with pH dropping below 6.5 resp. 6) and the average dwell time in days of the voice

**Fig. 3:**
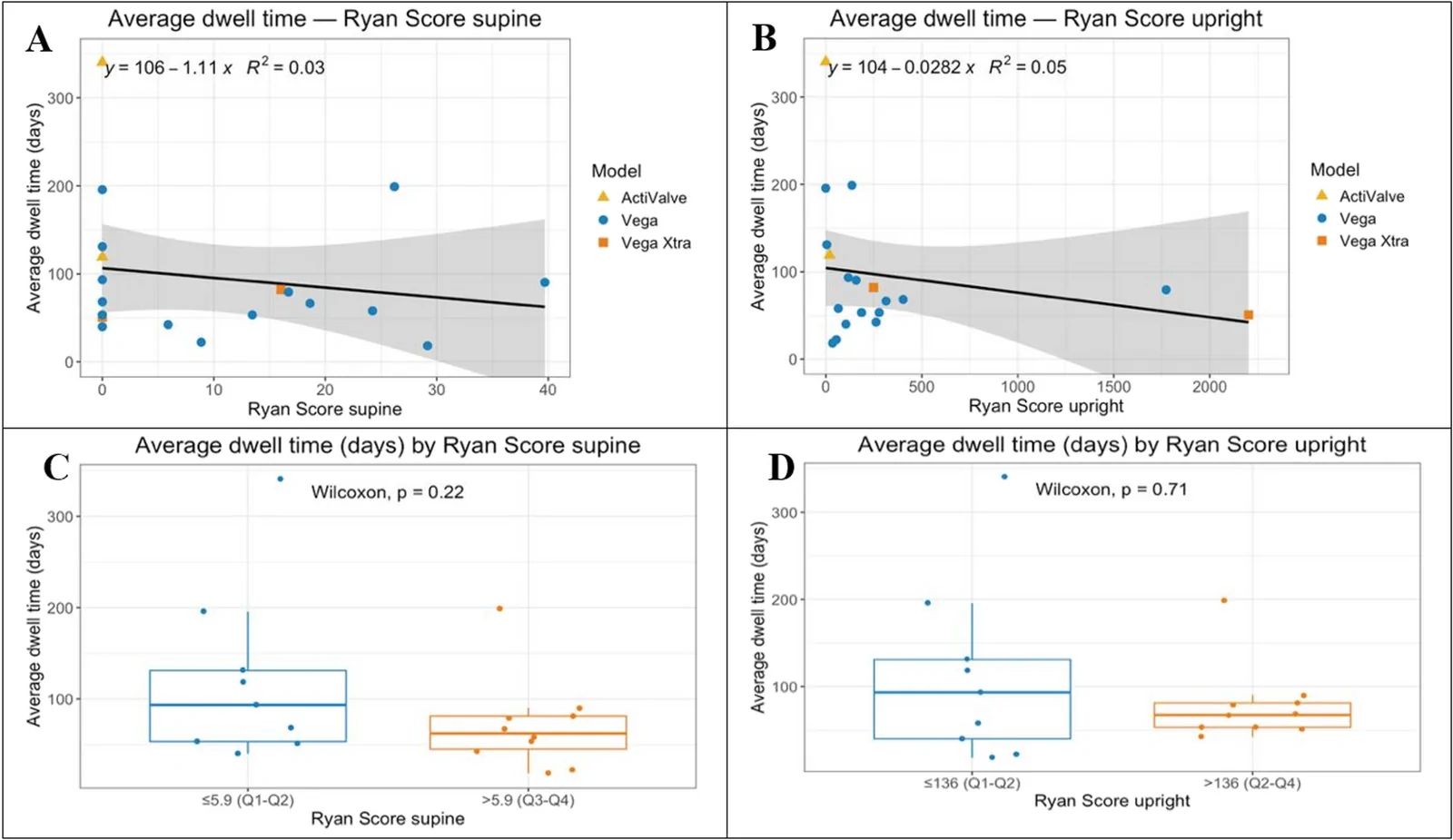
Our regression analysis could not reveal any clear correlation between the dwell time and the Ryan Score in supine and upright position

Regression analysis over all types of prostheses showed an inverse relationship between acid exposure (as assessed by the percentage of time in a 24-hour monitoring interval with pH dropping below 6.5 resp. 6) and the average dwell time in days of the voice prostheses (β = −1.85, 95% CI: −2.90 to −0.80, *p* = 0.0014, R2 = 0.40 for pH < 6.5 resp. β = −1.74, 95% CI: −2.95 to −0.52, *p* = 0.0071, R2 = 0.30 for pH < 6.0) as depicted in Fig. 2a and b. At lower pH thresholds, no significant associations with average dwell time were observed (pH < 5.5: β = −1.34, 95% CI: −2.90 to 0.22, *p* = 0.089, R² = 0.14; pH < 5.0: β = −0.77, 95% CI: −2.67 to 1.13, *p* = 0.41, R² = 0.03). Similarly, the Ryan score showed no significant association with average dwell time in either supine (β = −0.93, 95% CI: −4.01 to 2.14, *p* = 0.53, R² = 0.02) or upright position (β = −0.02, 95% CI: −0.09 to 0.03, *p* = 0.37, R² = 0.04), as depicted in Fig. 3a–d.

#### Vega-only subgroup

Because ActiValve^®^ and XtraSeal^®^ prostheses have inherently longer baseline dwell times compared to the standard Provox Vega^®^, a subgroup analysis restricted to Vega^®^ patients (*n* = 17) was performed to assess the consistency of this association in a device-homogeneous sample. This analysis confirmed the inverse relationship for pH < 6.5 (β = −1.32, 95% CI: −2.52 to − 0.12, *p* = 0.033, R² = 0.26; Fig. 2c). For pH < 6.0, a consistent negative trend was observed that did not reach statistical significance (β = −1.03, 95% CI: −2.32 to 0.26, *p* = 0.10, R² = 0.16; Fig. 2d).

#### Sensitivity analysis

Influence diagnostics identified one observation with Cook’s distance > 1, representing a clinically plausible value that was retained in the primary analysis (Supplementary Figure S1). The sensitivity analyses (Table 2) showed consistent results across all modeling approaches. The negative association between the percentage of time at pH < 6.5 and average dwell time remained significant in all models.

**Table 2:** Regression analysis

Model	*n*	coefficient	SE	95% CI	*p*-value	*R* ^2^
pH < 6.5, all (%) Standard OLS^a^ Robust regression^b^ Influential points excluded^c^ Log-transformed^d^	22 22 21 22	−1.85 −1.50 −1.04 −0.015	0.50 0.38 0.45 0.0051	−2.90, −0.80 −2.25, −0.76 −1.98, −0.09 −0.026, −0.0047	0.0014 0.0076 0.032 0.0069	0.40 0.37 0.21 0.31
pH < 6.0, all (%) Standard OLS^a^ Robust regression^b^ Influential points excluded^c^ Log-transformed^d^	22 22 21 22	−1.74 −1.29 −0.92 −0.014	0.58 0.46 0.48 0.0058	−2.95, −0.52 −2.20, −0.38 −1.93, 0.07 −0.026, −0.0017	0.0071 0.011 0.068 0.026	0.30 0.27 0.16 0.22

^a^Ordinary Least Squares regression, ^b^Calculated with Huber loss function, ^c^Cook’s D > 1 removed, ^d^Coefficient represents proportional change

##### Secondary outcome

**Fig. 4:**
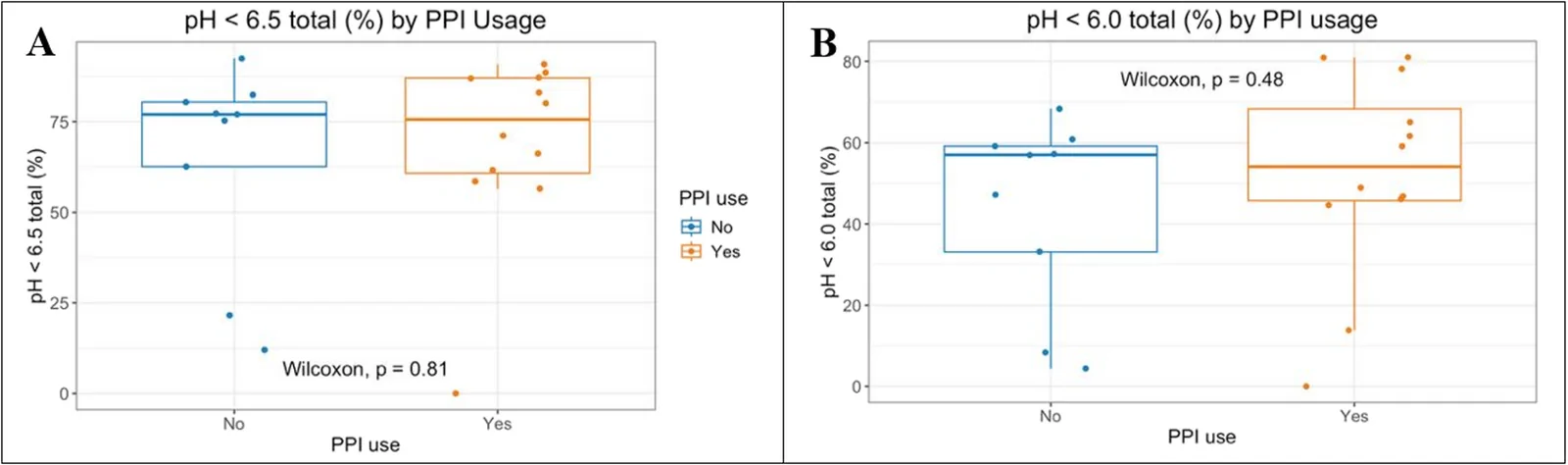
Box Plot showing no difference between PPI user vs. non PPI user in the acid exposure of the voice prostheses (as assessed by the percentage of time in a 24-hour monitoring interval with pH dropping below 6.5 resp. 6)

**Fig. 5:**
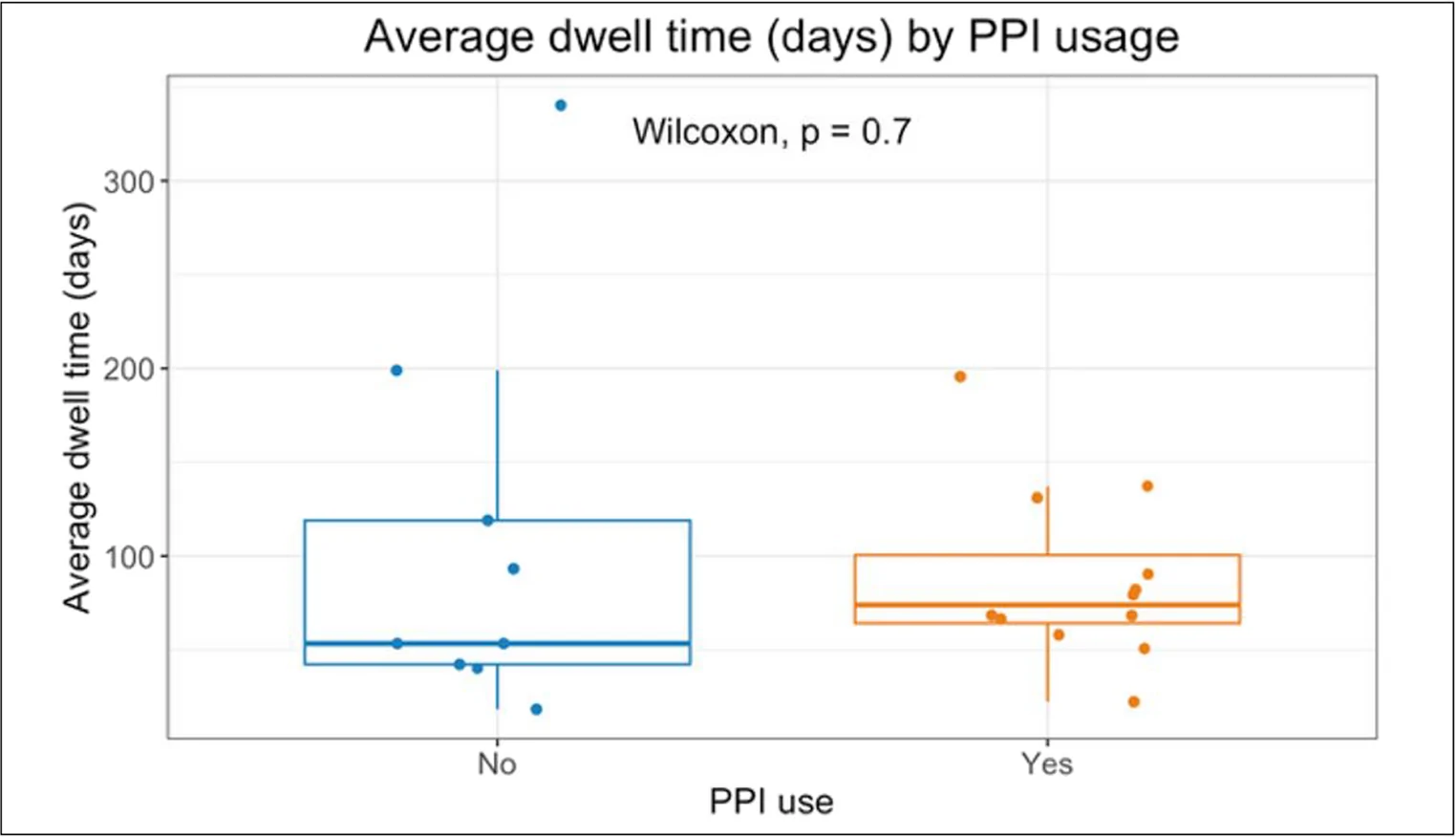
Box Plot showing no difference between PPI user vs. non PPI user in the dwell time of the voice in days

In our cohort, 12 patients were on regular PPI treatment that was not stopped during the pH monitoring study. Ten patients were off PPI. We analyzed whether patients on regular PPI treatment had less acid exposure and a longer dwell time of the provox vega voice prostheses compared to patients without PPIs. As seen in the box plot in Fig. 4a and b, patients with PPI usage did not show a significantly different acid exposure in the neopharynx at the level of the Provox^®^ voice prosthesis as assessed by the fraction of monitoring time with pH < 6.5 or pH < 6.0. Similarly, we did not see any significant difference in the dwell time between the two patients groups with and without regular PPI therapy as depicted in Fig. 5.

Screening of patient records did not reveal any relevant drug-drug interactions potentially reducing PPI efficacy. Pharmacogenetic testing was available for two patients on PPI, neither of whom carried the CYP2C19*17 allele associated with ultrarapid metabolism.

## Discussion

In our study, acid reflux exposure was inversely correlated with the dwell time of Provox^®^ voice prostheses. Proton pump inhibitor (PPI) treatment was not associated with a reduced acid exposure and prolonged dwell time of the voice prostheses, indicating insufficient effect of PPIs.

Only few studies explored the potential link between acid reflux and dwell time of voice prothesis after laryngectomy so far:

Hadzibegovic et al. reported no association of the “reflux marker” pepsin in the saliva with voice prosthesis complications. He also did not observe any effect of a 3 month treatment with PPI on voice prostheses complications [7]. The external validity of this study may be limited, as recent evidence show poor diagnostic accuracy of pepsin measurements in saliva as a surrogate marker for acid reflux [9, 11, 21].

In contrast, Lorenz et al. found a significant correlation between the extent and frequency of tracheoesophageal fistula complications and the severity of extraesophageal reflux measured by 24 h dual probe pH monitoring [13]. Similarly, Pattani et al. described an association of voice prosthesis complications such as voicing difficulties or granulation tissue with reflux [18]. In line with the latter data, our regression analysis showed an inverse relationship between acid exposure and dwell time as depicted in Fig. 2a to d. However, we did not find any correlation between the Ryan score and the dwell time of the voice prostheses (Fig. 3a - d).

The finding that acid exposure time at pH < 6.5 and < 6.0 was associated with dwell time, while the Ryan score and lower pH thresholds were not, might be explained by the predictor distribution in this cohort: at conventional thresholds (percentage of time with pH < 5.5, < 5.0), the majority of patients showed near-zero acid exposure time, resulting in a zero-inflated distribution that limits the statistical utility of standard linear regression at this sample size. The higher thresholds of pH < 6.5 and < 6.0 provided sufficient variability in the predictor to detect an association. Whether these thresholds are also the most clinically relevant for predicting prosthesis degradation in the post-laryngectomy setting remains to be determined in larger studies.

In the Vega-only subgroup (*n* = 17), the association for pH < 6.5 remained significant, while for pH < 6.0 a consistent negative trend was observed without reaching statistical significance. This might reflect reduced statistical power in the smaller subgroup rather than absence of a true association.

We were further interested if PPI treatment is associated with lower degree of acid reflux and a longer dwell time of the voice prostheses. In our study patients with PPI usage did not show a significantly different acid exposure in the neopharynx compared to patients without PPI (Fig. 4). That could also explain the missing difference of the dwell time of such voice prostheses in patients with and without PPI (Fig. 5). In line with our findings, a study by Lorenz et al. revealed pronounced acid reflux in most of the patients examined with pH monitoring post laryngectomy and rehabilitated with a voice prosthesis [14]. Furthermore, the study showed that only a smaller fraction of these patients responded to a PPI treatment with a neutralization of pH levels during the 24 h dual probe monitoring. Most patients, however, did only show either a partial or no response to PPI treatment in that study during 24 h dual probe monitoring [14]. In a different study following patients post laryngectomy with a 3-month PPI treatment, no reduction of voice prosthesis complications were observed [6].

Pharmacologically, PPI resistance may be explained by pharmacodynamic or pharmacokinetic mechanisms, or a combination of both [1, 3].

From a pharmacokinetic standpoint especially, reduced absorption or bioavailability, increased metabolism, or enhanced excretion may lead to decreased exposure to PPIs and thus reduced efficacy. Reduced absorption is typically observed when the enteric coating of the PPI is compromised prior to ingestion. In terms of metabolism, increased activity of CYP2C19, such as in individuals homozygous for the *17 allele (ultrarapid metabolisers) or heterozygous carriers of *17 and a normal-function allele (rapid metabolisers), often necessitates higher PPI doses to achieve adequate acid suppression. This is particularly relevant as most PPIs, with the exception of rabeprazole, are moderate to major substrates of CYP2C19 [1, 3, 8]. Novel variants, such as the CYP2C: TG haplotype (rs2860840 and rs11188059), may also reduce PPI exposure, although clinical evidence for these variants remains limited [8]. Known inducers of CYP2C19, such as rifampicin or apalutamide, should also be considered during medication review. However, none of the common factors were identified in this cohort. There are several limitations of our study: The use of a three-prosthesis average as a proxy for baseline dwell time may not fully capture intra-patient variability. Due to the limited sample size, multivariable adjustment was not feasible, and residual confounding cannot be excluded, although several key confounders, most notably radiotherapy (95%), were near-constant in this cohort. The inclusion of prosthesis types with different baseline dwell times may influence the all-prostheses estimates; the Vega-only subgroup analysis is presented to assess the robustness of this finding. The sample size is relatively small, so the missing effect of PPI might be explained by a type II error and a larger patient cohort is needed in future studies. Another explanation for the missing effect of PPI could be a selection bias with patients more prone to reflux symptoms being more likely on PPI treatment compared to patients without reflux symptoms.

Furthermore, Restech^®^ pH probes only assess the extent of acid reflux. Hence, other factors involved in the pathogenesis of extraesophageal/laryngopharyngeal reflux that could also affect the dwell time of voice prostheses such as digestive enzymes or bile acid are not assessed in our study [10, 11, 19]. Also the type and extent of the resection with disruption of reflux barriers as well as the type of flap/reconstruction might affect the extend of extraesophageal reflux, which needs to be addressed in future studies.

## Conclusion

The above findings and the available literature indicate an association of extraesophageal acid reflux with reduced dwell time of Provox^®^ voice prostheses and voice prosthesis complications. The effect of PPI treatment in suppressing extraesophageal acid reflux and in improving dwell time as well as complication rate of Provox^®^ voice prostheses remains unclear. Further studies are needed to elucidate the underlying mechanisms and to identify effective therapeutic strategies in prolonging voice prostheses dwell times.

## Supplementary Information

Below is the link to the electronic supplementary material.

Supplementary Material 1 (PDF 81.5 KB)

## Data Availability

Summary data supporting the findings of this study are available within the paper. Any further data are available from the corresponding author upon request
