# Supplementary material for: Inverse correlation between acid reflux and dwell time of voice protheses (Provox®) in laryngectomy/laryngopharyngectomy patients: a retrospective analysis of a prospective cohort
Source: Eur Arch Otorhinolaryngol. 2026 Jun 10;283(8):5161–8. doi: 10.1007/s00405-026-10370-1 (PMC13683532; doi:10.1007/s00405-026-10370-1)
Supplement: Supplementary file 1 [file 405_2026_10370_MOESM1_ESM.pdf]

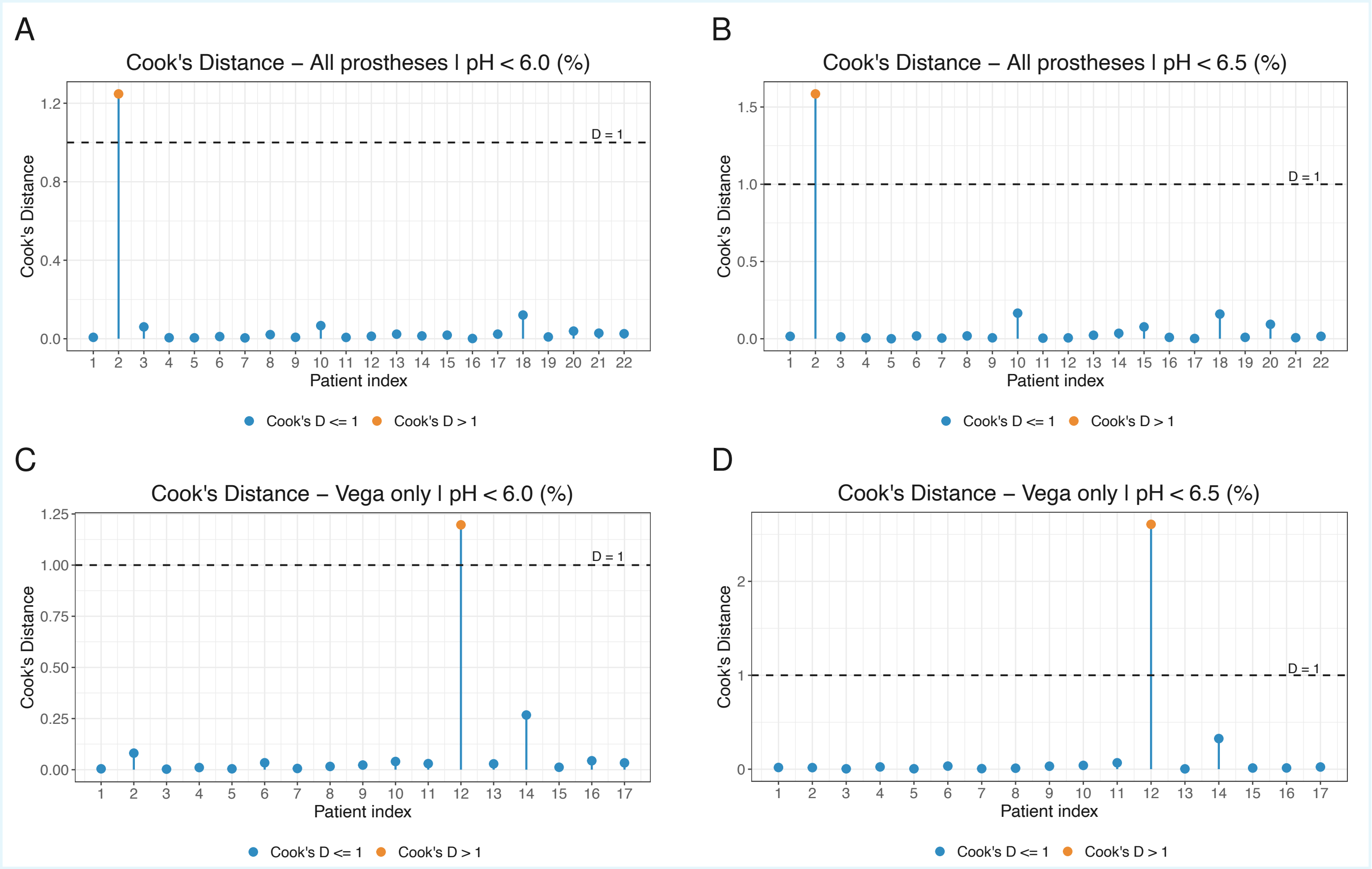

A
Cook's Distance – All prostheses | pH < 6.0 (%)
Cook's Distance
1.2
0.8
0.4
0.0
D = 1
1 2 3 4 5 6 7 8 9 10 11 12 13 14 15 16 17 18 19 20 21 22
Patient index
Cook's D <= 1
Cook's D > 1
B
Cook's Distance – All prostheses | pH < 6.5 (%)
Cook's Distance
1.5
1.0
0.5
0.0
D = 1
1 2 3 4 5 6 7 8 9 10 11 12 13 14 15 16 17 18 19 20 21 22
Patient index
Cook's D <= 1
Cook's D > 1
C
Cook's Distance – Vega only | pH < 6.0 (%)
Cook's Distance
1.25
1.00
0.75
0.50
0.25
0.00
D = 1
1 2 3 4 5 6 7 8 9 10 11 12 13 14 15 16 17
Patient index
Cook's D <= 1
Cook's D > 1
D
Cook's Distance – Vega only | pH < 6.5 (%)
Cook's Distance
2
1
0
D = 1
1 2 3 4 5 6 7 8 9 10 11 12 13 14 15 16 17
Patient index
Cook's D <= 1
Cook's D > 1
